# Functionalization of Ceramic Scaffolds with Exosomes from Bone Marrow Mesenchymal Stromal Cells for Bone Tissue Engineering

**DOI:** 10.3390/ijms25073826

**Published:** 2024-03-29

**Authors:** Ekaterina Maevskaia, Julien Guerrero, Chafik Ghayor, Indranil Bhattacharya, Franz E. Weber

**Affiliations:** 1 Center of Dental Medicine, Oral Biotechnology & Bioengineering, University of Zurich, Plattenstrasse 11, 8032 Zurich, Switzerlandjulien.guerrero@usz.ch (J.G.); chafik.ghayor@usz.ch (C.G.); indranil.bhattacharya@usz.ch (I.B.); 2 Center for Applied Biotechnology and Molecular Medicine (CABMM), University of Zurich, Winterthurerstrasse 190, 8057 Zurich, Switzerland

**Keywords:** bone tissue engineering, exosomes, extracellular vesicles, bone scaffold, bone marrow mesenchymal stromal cells, hydroxyapatite, tricalcium phosphate

## Abstract

The functionalization of bone substitutes with exosomes appears to be a promising technique to enhance bone tissue formation. This study investigates the potential of exosomes derived from bone marrow mesenchymal stromal cells (BMSCs) to improve bone healing and bone augmentation when incorporated into wide open-porous 3D-printed ceramic Gyroid scaffolds. We demonstrated the multipotent characteristics of BMSCs and characterized the extracted exosomes using nanoparticle tracking analysis and proteomic profiling. Through cell culture experimentation, we demonstrated that BMSC-derived exosomes possess the ability to attract cells and significantly facilitate their differentiation into the osteogenic lineage. Furthermore, we observed that scaffold architecture influences exosome release kinetics, with Gyroid scaffolds exhibiting slower release rates compared to Lattice scaffolds. Nevertheless, in vivo implantation did not show increased bone ingrowth in scaffolds loaded with exosomes, suggesting that the scaffold microarchitecture and material were already optimized for osteoconduction and bone augmentation. These findings highlight the lack of understanding about the optimal delivery of exosomes for osteoconduction and bone augmentation by advanced ceramic scaffolds.

## 1. Introduction

The shortage of tissue and organ donors remains one of the most significant challenges for global public health [1,2,3]. While autografts are the current gold standard for most transplantations [4,5,6], their use is constrained by several limitations, such as donor site morbidity and limited tissue availability [7]. The use of allografts, on the other side, is associated with such challenges as donor shortage for organ transplantations [8], severe immune rejection risks, and possible complications from infection or disease from the donor [9]. Therefore, the goal of tissue engineering is to develop fully functional synthetic substitutes for damaged tissues or organs that could replace auto- and allografts. To overcome the limited performance of synthetic scaffolds, they are often functionalized with living cells and/or growth factors [10,11].

The scaffold used as a substitute must meet several requirements to be used as a tissue-engineered graft. The perfect scaffold should satisfy the biological and mechanical requirements of the target tissue, have an appropriate microstructure and open-pore geometry to promote cell proliferation, migration, and differentiation of cells into the specific cell type, and exhibit suitable surface morphology [12,13]. As shown in our previous study [14], ceramic scaffolds based on triply periodic minimal surface designs are promising candidates for bone substitutes. To accelerate bone regeneration even further, cells or biologically active biomolecules can be integrated into these scaffolds [15].

Despite the high potential of stem cells to regenerate tissues, numerous problems such as tumorigenesis, fibrosis, and cell rejection are associated with their application [16]. Due to these challenges, the search for stem cell-free therapy is one of the current hot topics of research [17], and the functionalization of scaffolds with exosomes is a promising solution. Exosomes are small extracellular vesicles with a diameter ranging from 30 to 200 nm [18,19] that play an important role in cell-to-cell communication by delivering functional biomolecules, for example, microRNA, messenger RNA, proteins, and lipids [20]. Additionally, exosomes possess low immunogenicity and toxicity, they are biocompatible and stable [21], and they have less strict storage conditions compared to cells [22].

It was shown that exosomes improve postischemic neurological impairment very similarly to mesenchymal stromal cells [23], and in a rat myocardial infarction model, their beneficial effects were significantly superior compared to mesenchymal stromal cells [24]. Moreover, studies have demonstrated the ability of exosomes to stimulate chondrocytes, osteoblasts, osteoclasts, precursor cells, and immune cells, which are associated with bone and cartilage regeneration, to promote bone repair [17]. Furthermore, exosomes also have a higher biocompatibility and lower toxicity than synthetic nanomaterials [25].

Despite the high potential of exosomes in tissue engineering, numerous questions remain unanswered. Among them are the preferable source of exosomes, dosage, and duration of exosome treatment. The concentration and quantity of exosomes vary considerably between studies. In addition, there is no standard way of reporting researchers use of the amount of exosomes, which varies from 10^7^ to 10^13^ particles/mL [26,27,28,29]; volume of exosome solution, which is added to the cells or scaffolds (from 20 µL to 100 µL) [26,30]; amount of total protein of exosomes (10–100 µg) [31,32] or its concentration (50 µg/mL–1 mg/mL) [30,33,34]; or a combination of these values.

The choice of an exosome source depends on the application purposes, but even for bone tissue engineering, exosomes are isolated from different cells, including mesenchymal stromal cells derived from different tissues, adipocytes, dendritic cells, osteoclasts, osteoblasts, osteocytes, and monocytes [20]. Bone marrow mesenchymal stromal cells (BMSCs) have a multipotent nature, which includes their ability to differentiate into multiple cell types [35]. They possess immunomodulatory, repair, and pro-regeneration functions [36], which are capable of establishing a pro-regenerative microenvironment for injured tissue [37,38]. Additionally, BMSCs secrete bioactive molecules with pro-angiogenic, anti-fibrotic, chemotactic, and trophic properties, thus promoting tissue regeneration [35]. Furthermore, it was shown that BMSCs have higher osteogenic potential than mesenchymal stromal cells derived from adipose tissue due to the methylation status of the main transcription factors [39].

Given these considerations, our research aims to investigate the effects of BMSC-derived exosomes incorporated into ceramic scaffolds on cell growth, differentiation, and their potential to promote in vivo bone defect healing and bone augmentation.

## 2. Results

### 2.1. Characterization of Cells

**Figure 1:**
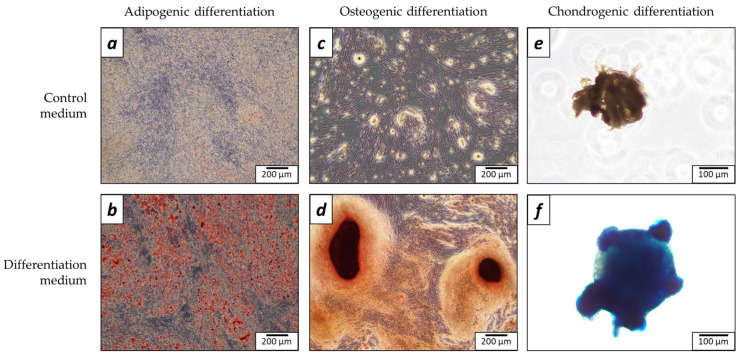
Images of trilineage differentiation of bone marrow mesenchymal stromal cells (BMSCs) after 21 days of culture. The upper row corresponds to cells cultured in the control medium and the lower row—in the differentiation medium. The staining was performed with Oil Red O for adipogenic differentiation (**a**,**b**), Alizarin Red for osteogenic differentiation (**c**,**d**), and Alcian Blue for chondrogenic differentiation (**e**,**f**). Scale bars are provided.

To prove the multipotent nature of the isolated BMSCs, their trilineage differentiation was performed (i.e., adipogenic, osteogenic, and chondrogenic). For the adipogenic lineage, numerous lipid droplets were visible on brightfield observation, even before the staining with Oil Red O solution in the differentiation group. In contrast, no lipid droplets were visible in the samples cultured within the control medium. The differences between the groups became even more noticeable after the staining (Figure 1a,b). For osteogenic differentiation, nuclei of mineralization appeared only in the presence of the osteogenic medium. The areas of mineralized matrix stained with Alizarin red can be seen on the osteogenic inductive samples, while no staining was visible in control samples (Figure 1c,d). The isolated BMSCs also differentiated into chondrocytic micromasse, as evidenced by Alcian blue staining (Figure 1e,f). As a result, BMSCs have demonstrated their multipotent phenotype by their ability for trilineage differentiation, the hallmark of mesenchymal stromal cells.

### 2.2. Nanoparticle Tracking Analysis (NTA)

**Figure 2:**
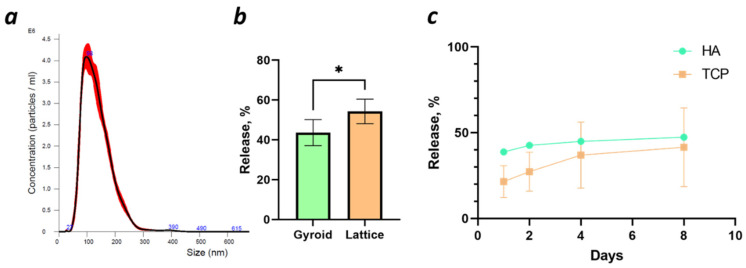
(**a**) Distribution of particles measured by Nanoparticle Tracking Analysis (NTA) (in red and mean value in black); (**b**) release of exosomes from the Gyroid and Lattice hydroxyapatite (HA) scaffolds after 24 h,* *p* < 0.05; (**c**) release of exosomes from the HA and tricalcium phosphate (TCP) scaffolds after 1, 2, 4, and 8 days.

The results of exosome measurements using NTA assay are presented in Figure 2a. The distribution is uniform with the highest concentration of particles with a diameter of 98 nm. We observed that 90% of particles were in the range of exosome sizes (30–200 nm). The concentration of exosomes was calculated, and we achieved 4.1 × 10^9^ ± 3.3 × 10^8^ particles/mL.

### 2.3. Proteomics

**Figure 3:**
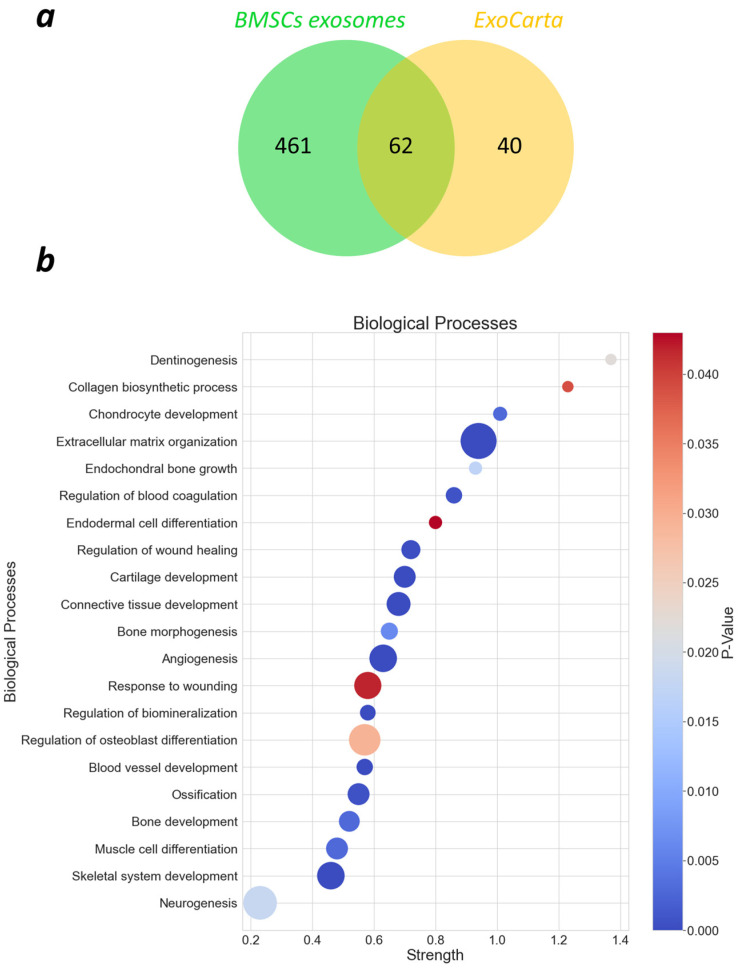
Results of proteomics analysis. (**a**) Venn diagram of exosomal proteins against ExoCarta; (**b**) biological processes Gene Ontology (GO) of the found proteins: color represents a *p*-value, and diameter of the bubbles is associated with an amount of proteins in the network, which are annotated with a particular term.

To gain insight into the exosomes’ composition, a proteomics study was performed, which revealed 523 different proteins derived from *Oryctolagus cuniculus*. These proteins were compared to the proteins associated with exosomes, which are listed on the ExoCarta website [40]. From our analyzed exosomes, 62 out of 102 proteins from ExoCarta were present in the protein pool (Figure 3a), which confirms that proteins were extracted from exosomes. Functional enrichment analysis was performed, and proteins were grouped by biological processes via Gene Ontology (GO) analysis (Figure 3b). We observed that, in total, 461 biological processes were associated with the proteins; however, to improve the clarity, only the most abundant and relevant are presented. Strength was calculated as Log10 (number of proteins in the network that are annotated with a term/number of proteins that we expect to be annotated with this term in a random network of the same size) and describes how large the enrichment effect was. Extracellular matrix organization was among the highly observed biological processes, although proteins associated with bone and cartilage development, angiogenesis, and wound healing were also highly expressed.

### 2.4. Migration

**Figure 4:**
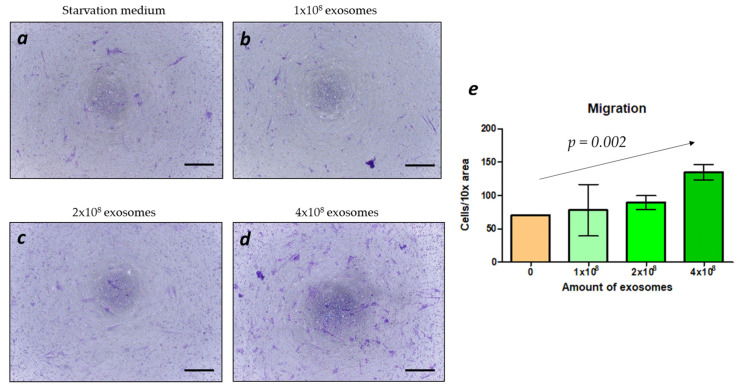
BMSCs in the amount of 6 × 10^4^ were cultured in the starvation medium without FBS in the upper part of the transwell insert; the lower part contained starvation medium without FBS (**a**) and the same medium with the addition of 1 × 10^8^ (**b**), 2 × 10^8^ (**c**), 4 × 10^8^ (**d**) of exosomes. Scale bars of 100 µm are provided; (**e**) quantification of the amount of the migrated cells. The *p*-value provided in (**e**) is the result of the Jonckheere–Terpstra trend test.

The highest cell migration was measured in the positive control groups (both FBS and exosome-free FBS). The supplementation of the starvation culture medium with exosomes also induced the migration of BMSCs (Figure 4a–d); the Jonckheere–Terpstra trend test revealed a significant correlation between the increase in BMSC migration and the increased amount of exosomes present in the culture medium (Figure 4e).

### 2.5. Release of Exosomes from the Scaffolds

The amount of exosomes released from the scaffolds was measured with the NTA. As presented in Figure 2b, a significantly higher amount of exosomes was released from the Lattice scaffolds. However, about 50% of exosomes stayed within scaffolds after 24 h.

Additionally, we measured exosome release over longer times within Gyroid scaffolds produced out of different materials (hydroxyapatite (HA) and tricalcium phosphate (TCP)), as these scaffolds were further used for the in vivo implantation. After 8 days, only 50% of exosomes were released from both ceramic-based scaffolds. It is noticeable that in the TCP group, the variance was increased, while the HA group showed more consistency (Figure 2c).

### 2.6. BMSC Metabolism and Differentiation

**Figure 5:**
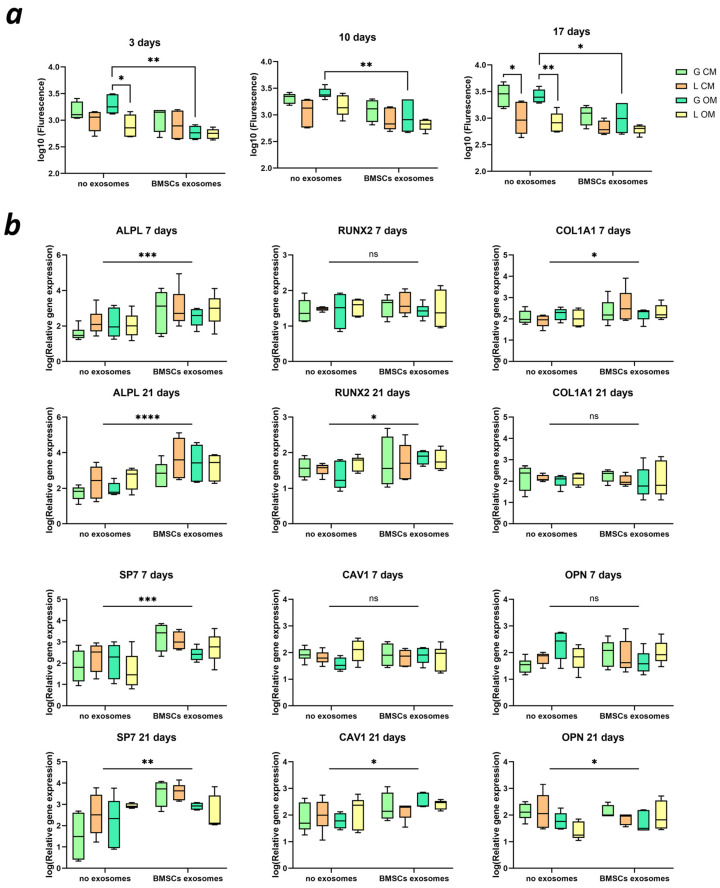
(**a**) Metabolism of BMSCs cultured within Gyroid (G) and Lattice (L) scaffolds in control (CM) and osteogenic (OM) medium, ns—not significant; * *p* < 0.05; ** *p* < 0.01; *** *p* < 0.001; **** *p* < 0.0001; (**b**) differentiation of BMSCs after 7 and 21 days of culture, tested for alkaline phosphatase (*ALPL*), Runx Family Transcription Factor 2 (*RUNX2*), collagen type I alpha 1 chain (*COL1A1*), Sp7 transcription factor (*SP7*), caveolin 1 (*CAV1*), and osteopontin (*OPN*).

We observed higher fluorescence as an equivalent of cell metabolism for BMSCs cultured within Gyroid scaffolds compared to Lattice scaffolds at 3, 10, and 17 days (Figure 5a). The predominance was more noticeable in the group without exosome addition. No differences between different culture media were visible, although the addition of exosomes led to a lower metabolism of BMSCs, especially for Gyroid scaffolds in the osteogenic medium. Results of the three-way ANOVA analysis with significance scores are presented in Table 1.

The osteogenic differentiation of BMSCs was quantified using RT-qPCR at 7 and 21 days (Figure 5b). Already at 7 days, the early markers of osteogenic differentiation, *ALPL*, *COL1A1*, and *SP7,* were significantly more expressed in cells cultured with the addition of exosomes. At 21 days, *RUNX2*, *CAV1,* and *OPN* were also overexpressed in the exosome group. No influence of scaffold microarchitecture was found. Results of the three-way ANOVA analysis with the significance scores between the groups with and without the addition of exosomes are presented in Figure 5b.

### 2.7. Implantation of Scaffolds with Exosomes

**Figure 6:**
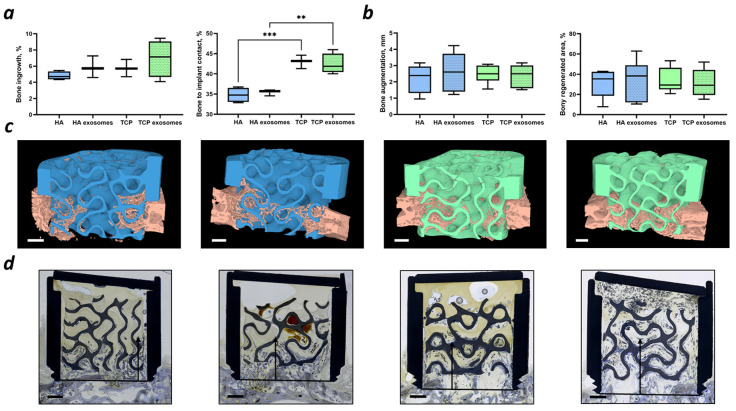
Quantification of (**a**) bone ingrowth and bone-to-implant contact for the defect model and (**b**) bone augmentation and bony regenerated area for the bone augmentation model, ** *p* < 0.01; *** *p* < 0.001; (**c**) µCT visualization of the ingrowth of the mineralized tissue (pink) into HA (blue) and TCP (green) scaffolds; (**d**) histological images of the bone ingrowth for the bone augmentation model; the arrows represent the distance of bone augmentation. The images are placed in the following order (from left to right): HA—HA with exosomes—TCP—TCP with exosomes. Scale bars of 1 mm are provided.

Only Gyroid scaffolds were implanted in two rabbit models, as they were associated with higher metabolism and slower exosome release, with no difference in BMSC differentiation. No influence of exosome addition to the ceramic-based scaffolds on bone ingrowth, bone-to-implant contact, or bone augmentation was detected (Figure 6). Nevertheless, the implantation of TCP scaffolds with and without exosomes was associated with a higher bone-to-implant contact compared to HA scaffolds. The histological and µCT images are provided.

## 3. Discussion

This study aimed to investigate the potential of exosomes derived from bone marrow mesenchymal stromal cells in enhancing the bone healing effects of highly osteoconductive ceramic scaffolds. BMSCs, known for their ability to differentiate into adipogenic, osteogenic, and chondrogenic lineages, were confirmed to possess the required multipotent nature, thus validating their suitability for exosome harvesting [41,42]. NTA is one of the most widely used techniques for quantifying extracellular vesicles [43] and is considered to be the most promising method [44]. Therefore, we used NTA for the identification and characterization of BMSC-derived exosomes in this study. This size-based methodology was supplemented by protein characterization. Together, we fulfilled the recommendations of the International Society for Extracellular Vesicles [45], since we used two different methods for exosome characterization and quantification, including a protein content-based exosome characterization. In our study, more than half of the proteins commonly associated with exosomes, which are listed on the ExoCarta website, were present in our protein pool, and 90% of the particles were in the size range of exosomes. Therefore, we could conclude that the extracted vesicles used in our study were, indeed, BMSC-derived exosomes.

As bioassays for our exosomes derived from BMSCs, we applied a migration test (Figure 4) and a BMSC differentiation test. The exosomes induced the migration of BMSCs in a concentration-dependent fashion, which is consistent with other studies [46,47]. Additional cell experiments including Alamar Blue assay and RT-qPCR (Figure 5) revealed lower metabolic activity of BMSCs cultured with exosomes, suggesting a higher osteogenic differentiation of BMSCs. Similar effects were reported by others [48,49]. Therefore, our exosomes were shown to be biologically active. However, no influence of the cell culture media on BMSC metabolism and differentiation was detected, which could be connected to the presence of the scaffold. Further experiments with undifferentiated and differentiated BMSCs without scaffolds should be performed to ensure that cell metabolism decreases upon differentiation. After the exosomes from BMSCs were characterized by size, protein content, and migration as bioassays, we applied them in vivo in combination with our highly osteoconductive ceramic scaffolds. Implantation experiments revealed a high bone ingrowth across all groups, irrespective of exosome supplementation. While exosomes have demonstrated efficacy both in vitro and in vivo by others [49,50,51], our findings suggest that the superior scaffold properties resulting from an improved microarchitecture and material composition make the supplementation with exosomes obsolete. Thus, our results indicate that scaffolds with enhanced properties may not require additional biologically active compounds such as exosomes to promote bone healing. A similar result was achieved with the addition of 10 µg of rhBMP-2 to 3D-printed TCP-based wide open-porous scaffolds [52]. For both bony bridging and bony regenerated areas, no significant improvement was achieved by the addition of 10 µg of rhBMP-2. One could hypothesize that by choosing a wide open-porous highly osteoconductive scaffold, as used here, the biology of bone healing of a non-critical size defect is already optimized, and further stimuli by MSC-derived exosomes or reasonable amounts of BMPs might be ill suited to enhance osteoconduction or bone augmentation any further.

Moreover, in vivo models showing a positive effect of exosomes on bone regeneration are mainly rodent-based [53,54]. The limitation of this research for small animal studies has already been reported, and further efforts are recommended to progress the research to large animal studies [55]. In our experiment, two in vivo rabbit models were performed. For BMP test systems, the efficiency of the growth factor decreases for bigger animals. Therefore, mouse and rat in vivo models represent initial model systems. Rabbit-based models are more challenging and, therefore, characterized as intermediate [56]. This could be the main reason why, in our two rabbit-based models, the addition of exosomes did not improve the measures significantly. For BMP, it meant that the effective dosage had to be increased from initial to intermediate for advanced models, and to unphysiological high numbers for humans [56]. That means that, in terms of animal models, further studies should focus on critical size defects for intermediate models where additional stimulation could be advantageous, as shown by others [55,57]. For further optimization and future clinical use, a fine-tuned release of exosomes by a carrier/delivery system appears mandatory [54,58]. One potential solution could involve the utilization of hydrogel scaffolds, known for their adjustable resorption rate and ability to regulate exosome release, as demonstrated in prior studies [17,59]. However, for critical size defects, hydrogels may prove inadequate due to their limited mechanical strength and rapid degradation [60]. Hence, they are suggested to be employed as a complement to ceramic scaffolds [61].

To improve regenerative outcomes, incorporating biological molecules, like BMP2 [62], mRNAs [63], or lncRNA [64], into exosomes could be explored. Research suggests that such constructs enhance angiogenic–osteogenic regeneration and prevent bone loss. Additionally, targeting exosomes to BMSCs through protein or aptamer integration may accelerate bone healing by accumulating them in the bone marrow [65,66]. These strategies hold promise for future investigations.

For our bone augmentation model, the delivery of exosomes might not be so crucial, since the construct is placed inside a titanium cylinder, which constrains exosome diffusion. Despite this diffusion barrier, exosomes enhanced neither the bone augmentation nor bony regenerated area. Since this is the first study on the efficiency of MSC-derived exosomes for bone augmentation, our results suggest that exosomes have no effect on bone augmentation.

In this study, we demonstrated the potential therapeutic efficacy of exosomes extracted from New Zealand white rabbits in promoting bone regeneration in syngeneic rabbit models. While our findings showcase promising results in a controlled setting, it is essential to consider the translational aspects of exosome-based therapies, particularly regarding their immunogenicity in non-syngeneic recipients. The importance of assessing immunogenicity in non-syngeneic rabbit models by evaluating immune responses in such models is crucial for understanding the broader applicability of exosome-based therapies in diverse patient populations. Therefore, future studies will aim to investigate the immunogenicity of these exosomes in non-syngeneic rabbit models.

Furthermore, extending our investigation to include non-syngeneic models will provide insights into potential immune reactions that may influence the safety and efficacy of exosome-based therapies in clinical settings. Addressing these aspects will be pivotal for advancing the development and eventual clinical translation of exosome-based regenerative therapies. Scaffold architecture also played a role in cell metabolism, although no differences in differentiation were found. This aligns with our prior findings, highlighting the superior characteristics of scaffolds based on triply periodic minimal surfaces [14]. Moreover, Gyroid scaffolds were shown to facilitate the slower release of exosomes, possibly due to their increased material and surface area, impacting solution distribution within the microporosity of the architecture. The scaffold material type also influenced exosome release kinetics, with scaffolds produced from TCP displaying a slower release rate compared to HA scaffolds. Since the microporosity accessible for liquid infiltration is 15% higher for TCP (Table 2), this difference in microporosity could fully account for the delayed exosome release in TCP compared to HA (Figure 2c). Therefore, sintering temperature-dependent microporosity could become a tool to tune exosome release if delivered in ceramics.

## 4. Materials and Methods

### 4.1. Extraction of BMSCs

BMSCs were extracted and isolated from rabbit tibias. After cleaning the bones from muscles and skin, they were disinfected using a 10% povidone-iodine solution (Betadine^®^). The tibias were cut and placed in 50 mL falcons with the extraction solution (PBS with the addition of 2% of 0.1 M EDTA and 1% of penicillin and streptomycin). After the centrifugation at 2000 rpm for 7.5 min, the released bone marrow was washed twice using the extraction solution and centrifuged at 1500 rpm for 5 min. Culture medium (αMEM (No. 22571-020) with the addition of 10% fetal bovine serum (No. 26140-079), 1% HEPES (1 M, No. 15630-056), 1% sodium pyruvate (100 mM, No. 11360-070), and 1% of penicillin and streptomycin (No. 15140-122) with L-glutamine (No. 25030-024) (all reagents were from Gibco, Schwerte, Germany) was added afterward together with 5 ng/mL FGF-2, and cells were seeded on gelatin-coated 75 cm^2^ tissue culture flasks. The medium was changed twice a week, and after the first passage, the cells were used in further experiments.

### 4.2. Characterization of BMSCs

To confirm the mesenchymal stromal cells’ multipotent nature, their trilineage differentiation was performed in accordance with existing protocols [69,70,71]. BMSCs were cultured either in a control or in a differentiation medium for 21 days with medium change twice a week. The pictures were taken after staining using the CKX53 optical microscope (Olympus, Tokyo, Japan).

#### 4.2.1. Adipogenic Differentiation

BMSCs were seeded in 12-well plates with a density of 1.5 × 10^4^ cells/cm^2^. The control medium consisted of DMEM (No. 31966021) with the addition of 10% fetal bovine serum (No. 26140-079) and 1% of penicillin and streptomycin (No. 15140-122) (all reagents were from Gibco, Switzerland). For the first four days, the induction medium consisted of the same components as the control medium with the addition of insulin (10 μg/mL; SLBZ8936), dexamethasone (1 μM; No. D2915), indomethacin (100 μM; No. I7378), and isobutylmethylxanthine (500 μM; No. I5879) (all reagents were from Sigma-Aldrich, Buchs, Switzerland). Further, the differentiation medium consisted of the same components as the control medium with the addition of 1 μg/mL of insulin.

For the staining, wells were washed with PBS, and the cells were fixed with 10% formalin for 5 min. After that, the formalin was changed to the fresh solution and incubated for 1 h. Afterward, cells were rinsed with 60% isopropanol and left to fully dry. Oil Red O solution was added for 10 min and discarded later; the cells were rinsed four times with distilled water and finally covered with PBS.

Oil Red O solution was prepared as follows: Oil Red O powder (No. O0625) was mixed with isopropanol in a concentration of 3.5 mg/mL by overnight stirring and filtered using a 0.2 µm filter. The prepared solution was mixed with the distilled water in a 6:4 relation and filtered one more time.

#### 4.2.2. Osteogenic Differentiation

BMSCs were seeded in 12-well plates with a density of 1.5 × 10^4^ cells/cm^2^. The control medium consisted of αMEM (No. 22571-020) with the addition of 10% fetal bovine serum (No. 26140-079), 1% HEPES (1M, No. 15630-056), 1% sodium pyruvate (100 mM, No. 11360–070), and 1% of penicillin and streptomycin (No. 15140-122) with L-glutamine (No. 25030-024) (all reagents were from Gibco, Switzerland). The osteogenic medium consisted of a control medium with the addition of 50 mM of L-ascorbic acid-2-phosphate (No. A8960), 10 mM of β-glycerophosphate (No. G9422), and 1 µM of dexamethasone (No. D2915) (all reagents were from Sigma-Aldrich, Switzerland).

For the staining, wells were washed with PBS, and the cells were fixed with 4% formalin for 15 min. Afterward, BMSCs were rinsed twice with distilled water, and 40 mM Alizarin red solution was added. After an incubation time of 30 min at room temperature with constant shaking, the cells were rinsed four times with distilled water and finally covered with PBS.

#### 4.2.3. Chondrogenic Differentiation

To form the cell pellets, 3 × 10^5^ cells were added to the 1 mL tubes and centrifuged for 5 min at 1500 rpm. The control medium consisted of DMEM (No. 31966021) with the addition of 1% of penicillin and streptomycin (No. 15140-122) (all reagents were from Gibco, Switzerland). The differentiation medium consisted of the same components as the control medium with the addition of TGFβ-3 (10 ng/mL; No. 8420-B3/CF, Bio-Techne, Minneapolis, MN, USA), dexamethasone (100 nM; No. D2915), 1% insulin + transferrin + selenium (ITS; No. I3146), and L-ascorbic acid-2-phosphate (1 µg/mL; No. A8960) (all reagents were from Sigma-Aldrich, Switzerland).

For the staining, the cells were washed with PBS and fixed with 10% formalin for 1 h. Afterward, cells were rinsed twice with distilled water and incubated overnight in Alcian Blue solution in the dark. After the incubation, the cells were washed twice with the destaining solution, consisting of 100% ethanol and acetic acid mixed in a 3:2 (*v*:*v*) ratio.

### 4.3. Extraction of Exosomes

In our study, non-differentiated BMSCs were cultured in 150 cm^2^ tissue culture flasks until 90% confluence. To extract exosomes, the cell monolayer was washed twice with PBS, and the medium was changed to the one with exosome-depleted FBS (No. A2720803, Thermo Fisher Scientific, Buchs, Switzerland). After 24 h, 20 mL of the medium was transferred to the 50 mL falcon, and 10 mL of the total exosome isolation reagent (No. 4478359, Invitrogen, Buchs, Switzerland) was added. After 24 h at 4 °C, the solution was centrifuged at 10,000 rpm and 4 °C for 1 h, and exosomes were resuspended in the desired volume of PBS. For all performed experiments, exosomes were stored at +4 °C for no longer than 1 week before their usage.

### 4.4. Characterization of Exosomes

#### 4.4.1. Nanoparticle Tracking Analysis (NTA)

The size distribution and concentration of exosomes were measured using NanoSight NS300 (Malvern Panalytical, Malvern, UK). Exosomes were diluted in PBS to have a concentration of 25 to 75 particles/frame. The solution was inserted in a 488 nm laser chamber with a constant flow rate, and five videos with a duration of 60 s each were recorded. NTA software (NTA 3.1 Build 3.1.54, Malvern Panalytical, Malvern, UK) was used to analyze the data, with a detection threshold of 5 and a camera level of 15. For the calculation of exosome concentration, only particles in a range from 30 to 200 µm were taken into account.

#### 4.4.2. Proteomics

Proteomics data acquisition was performed at Functional Genomics Center Zurich by LC-MS/MS DIA method with further processing using the Fragpipe v19 software. Further bioinformatics assay was performed with the use of UniProt, Gene Ontology, and STRING databases. Python v9 was used for the visualization of the results.

### 4.5. Migration

For the migration assay, THINCERT inserts with a pore size of 3 µm (No. 662630, Greiner Bio-One, Frickenhausen, Germany) were placed in the wells of a 24-well plate. Then, 6 × 10^4^ cells resuspended in 200 µL of the starvation medium (control medium without FBS) were added to the upper part (on the membrane). In the lower part, 500 µL of the starvation medium (control medium without FBS) was added for the negative control; it was supplemented with exosomes in an amount of 1 × 10^8^, 2 × 10^8^, and 4 × 10^8^ particles. For the positive control, 20% of FBS or exosome-free FBS was used instead of exosomes. The cells were placed in the incubator for 24 h; afterward, the staining of the migrated cells was performed as follows: both chambers were washed with PBS, and the filter was gently swabbed in the inside of each insert (upper part of the membrane) using cotton swabs. After the removal of non-migrated cells, remained cells were fixed with 4% formalin for 15 min. After the fixation, both chambers were washed with PBS, and 0.2% of crystal violet stain solution was added overnight. After the removal of the staining solution, the wells were washed with distilled water, and the remaining non-migrated cells were removed. The photos were taken using the CKX53 optical microscope (Olympus, Japan), and the number of migrated cells was calculated using ImageJ software (Version 1.54i).

### 4.6. Scaffold Production

Gyroid and Lattice scaffolds were designed using nTopology v.3 software (Version 3, USA) with a wall thickness of 0.2 mm and pores of 0.8 mm. Scaffolds were printed from hydroxyapatite (HA) and tricalcium phosphate (TCP) slurries (LithaBone HA 400 and LithaBone TCP 300) with the use of CeraFab 7500 (Lithoz, Vienna, Austria). The printed scaffolds were cleaned and sintered at 1300 °C and 1100 °C correspondently. The diameter of the scaffolds was 6 mm; for cell culture experiments, the height was 4 mm; for implantation, the height was 5 mm, and an additional ring with 2.5 mm in height and width was added to the top part to prevent the scaffold from falling into a defect.

### 4.7. Culture of BMSCs with Exosomes

To study the influence of exosome addition on the BMSC metabolism and differentiation, cell culture experiments were performed. HA-based Gyroid and Lattice scaffolds were placed into the wells of 24-well plates, which were preliminarily coated with 2% agarose solution to prevent cell attachment to the plates instead of the scaffold surface. Exosomes in a concentration of 4.1 × 10^9^ ± 3.3 × 10^8^ particles/mL diluted in 50 µL of PBS were added to the scaffolds of the corresponding group. After 1 h, BMSCs diluted in 50 µL of medium were seeded in the concentration of 10^6^ cells/scaffold. After 1 h, 900 µL of control or osteogenic (with the addition of 50 μM of ascorbic acid, 10 mM of β-glycerophosphate, and 1 µM of dexamethasone) medium with the exosome-depleted FBS was added. Cells were cultured for 21 days under a 5% CO_2_ atmosphere at 37 °C, and medium was changed twice a week.

Metabolism measurements were performed once a week with the Alamar Blue assay. Resazurin sodium salt (No. R7017-1G, Sigma-Aldrich, Switzerland) was preliminary diluted in PBS at a concentration of 50 µM and mixed for the measurement with a control medium at a concentration of 10%. The culture medium already in contact with the cells was changed to the prepared solution, and BMSCs were incubated for 4 h at 37 °C. Afterward, 150 µL of the solution was transferred to the 96-well plate, and its fluorescence was measured with the Synergy HT spectrophotometer (Agilent, Santa Clara, CA, USA) at an excitation wavelength of 530 nm and an emission wavelength of 590 nm.

Differentiation of BMSCs was studied using RT-qPCR. To extract RNA, scaffolds with BMSCs were destroyed after 7 and 21 days of culture using tissue lyser (QIAGEN, Venlo, The Netherlands) in the presence of QIAzol lysis reagent (QIAGEN, Germany), and then RNeasy Plus Mini kit (No. 74034, QIAGEN, Germany) was used. cDNA was obtained with the use of iScript cDNA Synthesis Kit (No. 1708891, Bio-Rad, Feldkirchen, Germany) with the Mastercycler gradient (Eppendorf, Hamburg, Germany) and mixed with the iTaq Universal SYBR Green Supermix (No. 1725124) and the primers for the following genes (Bio-Rad, Switzerland): *ALPL*, *RUNX2*, *COL1A1*, *SP7*, *OPN*, *CAV1* (osteogenesis-related genes), *GAPDH*, and *18S* (reference genes). RT-qPCR was performed with the use of CFX Connect (Bio-Rad, Switzerland), followed by the determination of the relative gene expression by the 2^−ΔΔCt^ method. The data were normalized to the expression of both reference genes at day 0.

### 4.8. Release of Exosomes from the Scaffolds

To study the effect of the microarchitecture on the short-term release of exosomes, 50 µL of exosomes was added to the HA-based Gyroid and Lattice scaffolds and placed in the wells of a 24-well plate. After 1 h, 950 µL of PBS was added to the wells. After 24 h, the solution of PBS with the released exosomes was measured by NTA to calculate the number of particles.

To study the effect of the material, a further long-term experiment was performed with the Gyroid architecture. HA and TCP scaffolds were placed into wells of 24-well plate, and 30 µL of exosomes was added to them. After 1 h, 970 µL of PBS was added. The solution was transferred to the tubes for further measurements after 1, 2, 4, and 8 days, and 1 mL of fresh PBS was added to the scaffolds.

Exosomes were also added to the wells without scaffolds to take into account the possible attachment of exosomes to the plastic surface.

### 4.9. Implantation of Scaffolds

For the implantation experiments, Gyroid scaffolds were produced from TCP and HA. For the group with exosomes, 30 µL of exosomes was added to the scaffold with a concentration of 1.8 × 10^10^ ± 1.3 × 10^9^ exosomes/mL or 5.3 × 10^8^ ± 3.8 × 10^7^ exosomes/scaffold. Two types of experiments were performed: bone defect and bone augmentation. All animal procedures were approved by the Animal Ethics Committee of the local authorities (Canton Zurich, 065/2018 and 090/2021) and performed following the ethics criteria contained in the bylaws of the Institutional Animal Care and Use Committee. The procedure was carried out as described earlier [72]. Briefly, four calvarial defects were made per animal (female, 26-week-old, New Zealand white rabbit). For bone augmentation, four circular slits were created in the calvarium of an animal, and four titanium cylinders filled with the scaffolds were screwed into the prepared slits and closed with a titanium lid to allow bone ingrowth only in one direction. To avoid the influence of the scaffold position on the results, sample assignment to its placement varied from animal to animal. The experiments lasted for four weeks, and, after that, the animals were anesthetized and sacrificed using an overdose of pentobarbital.

Afterward, samples were polymerized in methyl methacrylate solution and scanned with µCT using SkyScan 1272 (Bruker, Belgium) with the following parameters: Al 0.5 + Cu 0.038 filter, voltage of 80 kV, current of 125 µA, pixel size of 10 µm, and rotation step of 0.3°. NRecon software (software in Bruker MicroCT 3D Suite from 2022) was used for reconstruction, and DataViewer and CTVox (software in Bruker MicroCT 3D Suite from 2022) were used for the visualization of the scans. CTAn software (software in Bruker MicroCT 3D Suite from 2022) was used for the quantification of bone ingrowth inside the scaffolds, for which the region of interest (ROI) was defined as the inner part of the scaffold without the outer ring or the surrounding tissue. Analysis of bone tissue ingrowth was made with the same thresholding for all scans and normalized to the volume of ROI. For the bone-to-implant contact study, the volume of mineralized tissue was measured in the area close to the inner part of the scaffold’s surface at a distance of three pixels (30 µm). All software was provided by Bruker (Billerica, MA, USA). Due to the titanium shell, we were not able to perform the µCT scanning of the bone augmentation samples, and such parameters as bone augmentation and bony regenerated area were calculated using their histological images, which were made after cutting the samples in the middle of the defect and staining with Toluidine Blue.

### 4.10. Statistical Analysis

Data analysis was performed using GraphPad Prism v.9 (GraphPad Software, La Jolla, CA, USA). A normalization test was performed in each case, and a parametric or nonparametric analysis was selected in accordance. For the comparison of two independent groups, the Mann–Whitney test was used, and for several groups in other experiments, the Kruskal–Wallis test followed by Dunn’s multiple comparison tests was applied. For the three parametrical analyses, a three-way ANOVA was used, followed by Sidak multiple comparison tests. Additionally, the Jonckheere–Terpstra trend test [73] was used to evaluate the trend for exosome amount in the migration study. Results were considered significant at *p* < 0.05.

## 5. Conclusions

In conclusion, BMSC-derived exosomes exhibit potential in fostering bone tissue development, as evidenced by their ability to attract cells and enhance their differentiation into the osteogenic lineage. This assumption is further supported by our proteomic analysis, which identified proteins associated with bone formation. Unexpectedly, these exosomes delivered by wide open-porous Gyroid scaffolds had no effect on the healing of a non-critical size defect nor bone augmentation. In the future, more challenging defect models must be applied, as well as the release and number of exosomes tuned to affect bone healing. For bone augmentation purposes, the efficiency of exosomes is still not proven.

## Figures and Tables

**Table 1:** Results of 3-way ANOVA analysis of the metabolism study, ** *p* < 0.01, *** *p* < 0.001, **** *p* < 0.0001, ns—not significant.

Source of Variation	3 Days	10 Days	17 Days
Exosomes	***	****	***
Scaffold	**	**	****
Medium	ns	ns	ns

**Table 2:** Microporosity of the HA and TCP scaffolds.

	Peak Sinter Temperature, °C	Micropore Diameter, µm	Microporosity by Infiltration, %	Reference
HA	1300	1.21 ± 0.50	17.79 ± 0.78	[67]
TCP	1100	0.70 ± 0.24	21.41 ± 0.45	[68]

## Data Availability

Data are available upon request.
